# Editorial: The role of adipose tissue and resident immune cells in infections

**DOI:** 10.3389/fimmu.2024.1360262

**Published:** 2024-02-23

**Authors:** Luzia Teixeira, Jason K. Whitmire, Christine Bourgeois

**Affiliations:** ^1^ UMIB-Unidade Multidisciplinar de Investigação Biomédica, ICBAS-Instituto de Ciências Biomédicas Abel Salazar, Universidade do Porto, Porto, Portugal; ^2^ ITR-Laboratory for Integrative and Translational Research in Population Health, Porto, Portugal; ^3^ Department of Genetics, University of North Carolina at Chapel Hill, Chapel Hill, NC, United States; ^4^ Université Paris-Saclay, French National Institute of Health and Medical Research (INSERM), French Alternative Energies and Atomic Energy Commission (CEA), Center for Immunology of Viral, Auto-immune, Hematological and Bacterial diseases (IMVA-HB/IDMIT), UMR1184, Le Kremlin Bicêtre, France

**Keywords:** adipose tissue, infection, adaptive immunity, innate immune cells, metabolism, diet-induced obesity

Adipose tissues are distributed throughout the body and are in direct contact with sites of infection entry, such as mucosal tissues, gut, and skin (1). There is increasing appreciation that adipose tissue can influence local and systemic immune responses to infection (2). The adipose tissue can modulate immune responses through changes in the expression of pro- and anti-inflammatory cytokines, adipokines, and hormones that regulate general metabolism. Immune cells within adipose show differentiation states that are distinct from those in circulation or in lymphoid tissues, suggesting that the adipose environment regulates immune cell activity (3). The mechanisms leading to immune cell recruitment and maintenance within adipose remain enigmatic. Changes in diet that lead to obesity can alter the frequencies of regulatory or effector cells within adipose (4, 5), potentially affecting local and systemic immune protection. Obesity is associated with higher susceptibility to infections possibly due to alterations in immune cells residing in adipose tissue (6, 7). The interplay between immune cells and adipose is complex and bi-directional: immune cells residing within adipose tissue can perturb adipose tissue homeostasis and whole-body metabolism (2) and, conversely, metabolic cues can modulate immune responses (8). Several reports demonstrate that microorganisms can target and even persist in adipose tissues (9, 10). However, relatively few studies have addressed the immunological consequences of adipose tissue infection.

The aim of this Research Topic was to provide further information about the immune cells present in adipose tissue and their contribution to host resistance or susceptibility to infection. In this emerging field, four contributing papers discuss how adipose tissue can affect host defenses against pathogens, including at barrier sites, and identify a relationship between innate responses to the microbiome and myeloid cell activity at distal sites.

In a review article, entitled “*Beyond energy balance regulation: The underestimated role of adipose tissues in host defense against pathogens*”, Barthelemy et al. discuss pathogens (bacteria, parasite, and virus) that have been found in adipose tissue and describe infection-induced changes in immune cell populations present in this tissue. Emphasis is given to studies characterizing pathogen-specific memory T cells in adipose tissue, which may be important for resistance to infection. Not only are immune cells impacted by infection, but adipocyte metabolism can be targeted by pathogens, resulting in alterations in adipokine levels and lipid metabolism that can affect whole body metabolism. Several examples are mentioned with a particular emphasis on respiratory viruses Influenza A and SARS-CoV-2 that can also target adipose tissue. This review highlights the central role of adipose tissue where pathogens and immune cells coexist.

Adipocytes are present in skin and make factors that support skin integrity and affect immune protection at that site. In “*Skin-associated adipocytes in skin barrier immunity: a mini-review*”, Guan et al. describe the relationships between skin-associated adipocytes and how they influence immune defenses against infections. The review describes adipocyte production of pro-inflammatory adipokines, including leptin, chemerin, visfatin, and anti-inflammatory adipokines, including adiponectin and C1q/TNF-related protein-3. There is a discussion of skin adipocyte production of anti-microbial peptides, such as cathelicidin and LL-37, and a description of how skin-associated adipocytes make leptin that induces keratinocyte production of beta-defensin-2. Similarly, adipocyte expression of visfatin can induce cathelicidin, S100A7, BD-2, BD-3 from keratinocytes. The mini-review describes how skin-associated adipocytes produce chemerin, which can have direct antibacterial effects. Overall, this mini-review highlights how skin-associated adipocytes produce cytokines, adipokines, and anti-microbial peptides to support skin barrier immunity, and more generally, how this may impact inflammatory skin diseases, such as atopic dermatitis and psoriasis.

Complications due to metabolic diseases have emerged as a leading cause of mortality among persons with HIV worldwide. In an original research article, entitled “*Changes in subcutaneous white adipose tissue cellular composition and molecular programs underlie glucose intolerance in persons with HIV*”, Bailin et al. provide a comprehensive study of the adipose tissue cellular environment present in varying degrees of metabolic alterations in persons living with HIV (PLWH). This article describes changes in transcriptomic profile and intercellular communication in relation to glucose intolerance in PLWH. A coordinated intercellular regulatory program that enriched for genes related to inflammation and lipid-processing emerged across multiple cell types (T cells, macrophages, stromal cells) as glucose intolerance increased. This article reinforces the notion of the close connection between immune and metabolic cells in adipose tissue. Whether these mechanisms resemble those observed in non-HIV infected individuals remains to be further investigated, although some specific intercellular communication pathways are already suggested in the current work.

Microbiota can affect the immune cells regionally and systemically. In an original research article, entitled “*Gut REG3γ-associated Lactobacillus induces anti-inflammatory macrophages to maintain adipose tissue homeostasis*”, Huang et al. examined how species of intestinal bacteria can alter the frequencies of anti-inflammatory macrophage populations in the small intestinal lamina propria, as well as in the spleen and adipose tissues. The authors compared macrophage responses in wild-type (WT) mice or in transgenic mice that over-express human REG3γ, a bactericidal factor made by gut epithelial cells. The authors find that the gram-positive bacteria, *Lactobacillus*, including a strain*, NK318.1*, are enriched in the gut microbiota of mice overexpressing the antimicrobial peptide REG3γ. When *Lactobacillus NK318.1* is delivered orally to WT mice, there was an increased proportion of macrophages in lamina propria, spleen and adipose tissue with an anti-inflammatory profile (F4/80^+^ IL-10^+^ cells). Interestingly, oral gavage with *Lactobacillus NK318.1*, or the adoptive transfer of macrophages isolated from the lamina propria of *Lactobacillus*-infused mice, prevented high-fat diet induced obesity in recipient mice. The transferred macrophages from the *Lactobacillus*-infused mice migrated to the adipose tissue of recipient mice, and the authors suggest these gut macrophages support adipose tissue homeostasis.

The collection of articles in this Research Topic emphasize the importance of understanding the crosstalk between immune and non-immune cells residing in adipose tissue. These interactions can affect local or systemic immune defenses against pathogens. The metabolism of immune cells and adipose are impacted by these interactions and change in the context of infection and diet-induced obesity. A deeper understanding of immune-adipose tissue crosstalk in the context of infection is needed to develop therapeutic strategies to improve resistance to infection and maintain adipose tissue homeostasis.

## Author contributions

LT: Writing – original draft, Writing – review & editing. JW: Writing – original draft, Writing – review & editing. CB: Writing – original draft, Writing – review & editing.
